# Likelihood-based inferences for active-arm trial with counterfactual incidence based on recency assay

**DOI:** 10.1515/scid-2023-0004

**Published:** 2024-05-08

**Authors:** Yongwu Shao, Fei Gao

**Affiliations:** Gilead Sciences, Foster City, CA, USA; Vaccine and Infectious Disease Division, Fred Hutchinson Cancer Center, Seattle, WA, USA

**Keywords:** HIV PrEP, recency assay, likelihood ratio test, HIV incidence rate

## Abstract

**Objectives:**

The approach of using HIV recency assay to estimate the counterfactual incidence rate is being used as the primary efficacy method in a few ongoing large-scale HIV pre-exposure prophylaxis (PrEP) trials, and the current available approach for the inference is based on the Wald method that leverages the asymptotic distribution of the estimators. One issue with the Wald test is that it does not work well when the number of HIV infections are small in the active arm, and it fails to work when there are zero HIV infections. As future long-acting PrEP products are becoming more efficacious, it is very likely that a small or zero number of infections will be observed in HIV prevention trials, especially for subgroup analyses or interim analyses, hence there is a pressing need to develop inference methods that work under such scenarios.

**Methods:**

It is well known that when the sample size is small to moderate, likelihood ratio tests are more reliable than Wald tests in terms of actual error probabilities coming close to matching nominal levels. In this manuscript we derive the likelihood ratio test and the likelihood-based confidence intervals for HIV prevention trials based on recency assays.

**Results:**

Compared with the Wald test, the proposed method works when there are zero infections. Additionally, unlike the Wald test, the p-value from the likelihood ratio test is an increasing function with respect to the number of infections, which is a desirable property as otherwise it will cause confusions.

**Conclusions:**

For HIV PrEP trials based on recency assay, the likelihood-based p-value and confidence interval can be preferable to the Wald based inference methods when the number of HIV infections is expected to be small.

## Introduction

Since the first HIV pre-exposure prophylaxis (PrEP) product, Tenofovir disoproxil fumarate with emtricitabine (TDF/FTC), got approved for prevention by the US Food and Drug Administration (FDA) in 2012, an increasing number of PrEP agents are becoming available, for example, tenofovir alafenamide with emtricitabine (TAF/FTC) was approved in 2019, and long-acting cabotegravir was approved in 2021. Despite of these highly effective PrEP agents, new infections remain unacceptably high in many regions and subpopulations. Therefore, there is still an urgent need for effective, safe, and accessible HIV prevention options among at-risk populations.

It is recognized that it may not be feasible to conduct fully powered active-control non-inferiority trials for future PrEP products [1] as the required sample size would be too large. To address this issue, the Forum for Collaborative Research (the Forum) established the Recency Assay Working Group and published a consensus statement recommending the use of HIV recency infection assays for estimating HIV incidence in people not on PrEP as an external counterfactual to which on-PrEP incidence in trial subjects can be compared [2]. The Forum is a public-private partnership, brings together all stakeholders in a neutral and independent venue for open dialogue and deliberation. Individuals who participated in this project brought expertise from patients, at-risk community organizations, regulatory agencies, World Health Organization (WHO), regulated industries, and HIV prevention clinical science and research organizations [2]. Currently, a few ongoing HIV PrEP studies are employing this strategy as the primary analysis method, for example, PURPOSE-1 (NCT04994509, planned n=5,000) and PURPOSE-2 (NCT04925752, planned n=3,000) for the evaluation of lenacapavir. The primary efficacy analysis of the IMPOWER 022 trial (NCT04644029, planned n=4,500, for the evaluation of islatravir) was also planned to be based on this strategy, although the study was stopped early in 2022 for safety reasons.

In the Forum’s consensus statement, the recommended method for the background HIV incidence rate is the Kassanjee’s formula [3], and the sample size and the inference methods are to be based on Gao et al. [4]. Specifically, asymptotic distributions of the log estimated incidences are combined to derive the asymptotic distribution of the test statistic under null and alternative hypotheses, allowing for calculation of the sample size with desired type-1 error and power under specific null and alternative settings of intervention efficacy [2]. The assumptions underlying the Kassanjee’s formula are detailed in Gao and Bannick [5]. Gao’s method (based on the log scale) is also being used in observational studies such as the SIENA trial [6] and the evaluation of the HIV recency samples in the ECHO trial [7].

The test statistic in Gao et al. [4] is based on the Wald test, which leverages the asymptotic normal distribution of the Wald test statistic in large samples. However, current ARV-based PrEP products are highly efficacious and HIV infections are expected to be rare in the trials. In the HIV Prevention Trials Network (HPTN) 084 trial [8], for example, only 4 infections were observed among the 1,614 subjects in the Cabotegravir arm. The Wald method may not perform well when there are few events in the active arm of the trial. Specifically, we found that the p-value from the Wald method may decrease when the number of HIV infections on PrEP increases. For example, for a hypothetic HIV PrEP trial conducted where the dominant HIV subtype is Subtype C, assume 3,000 participants are screened for HIV, 5 % (150) of them are HIV positive and get the recency test, and 15 of them are deemed as recent infections by the Recent Infection Testing Algorithm (RITA). Suppose in the subgroup analysis or the interim analysis of the trial, one HIV infection is observed for a total follow-up time of 3,000 person years. Assume we use the assay parameters for HIV Subtype C reported in Kassanjee et al. [9] with a viral load cutoff of 75 copies/mL. Assume the null hypothesis is that the risk ratio is ≥0.80. Then the one-sided p-value by the Wald test is 0.0007. If we increase the number of infections on PrEP from one to two, we expect the p-value to increase as the observed prevention effect is getting worse. However, the Wald p-value actually decreases from 0.0007 to 0.0003 (see Figure 7). These observations strongly indicate that the Wald method may not be appropriate to use when there are few HIV infections in the trial.

Another issue with the Wald test is that it fails to give a p-value when there are zero HIV infections in the trial, which has a non-negligible chance for subgroup analyses or interim analyses as future long-acting PrEP products are expected to be highly efficacious. For example, if the actual incidence rate in the trial is 0.2/100PY (observed in the Cabotegravir arm of the HPTN 084 study), during the interim analysis 1,500 person years of follow-up time are accumulated, and the number of HIV infection is Poisson-distributed, then the chance of observing zero infections at the interim analysis will be 5 %, which cannot be ignored in practice.

In this manuscript we propose to use the likelihood ratio test instead of the Wald test for the HIV PrEP trials based on the recency assays. It is well known that when the sample size is small to moderate, likelihood ratio tests are more reliable than Wald tests in terms of actual error probabilities coming close to matching nominal levels [10]. The SAS user manual for the GLIMMIX procedure (https://documentation.sas.com/doc/en/statug/15.2/statug_glimmix_details31.htm) states that ‘the likelihood ratio test is almost always preferable to the Wald test, unless computational demands make it impractical to refit the model.’ Although the statement is in the context in the generalized linear mixed effect model, the concept applies to other models as well. In this manuscript we show that the proposed likelihood-based method works even when there are zero HIV infections in the intervention phase, and it is more reliable than the Wald-based method when the number of events in the trial is small.

## General setup

In this manuscript we use the same notations and set-up as in Gao et al., except that in Gao et al., it is assumed that all the HIV-positive subjects will take the recency test, while in this manuscript we assume only a portion of them will take the recency test, to address the possible scenario of missing samples, for example. Specifically, the HIV test will be applied to all volunteers who were screened for the PrEP trial, to estimate the HIV incidence that serves as external counterfactual. Table 1 gives the commonly used notations that will also be formally introduced in the next sections.

### Kassanjee’s estimator for the cross-sectional HIV incidence

Suppose that we screen N volunteers, not taking PrEP, for a clinical trial of a candidate PrEP agent. Each person is screened for HIV infection at the screening time (time 0). Those who are found to be HIV-positive at time 0 are assessed through a RITA to determine whether the HIV infection is “recent”, i.e., with a duration of at most T prior to screening. Here T is a pre-specified cut-off time for indicating a relevant time span for incidence estimation which is usually set to be 2 years. Suppose that at time 0, $N_{+}$ subjects are HIV-positive and $N_{-}=N-N_{+}$ are HIV-negative. Suppose that among the $N_{+}$ HIV-positive subjects, a random subset of $N_{\text{test}}$ subjects take the recency test, i.e., $N_{\text{test}}∼Binom\left(N_{+},q\right)$, where q is the probability of an HIV positive subject to take the recency test. Suppose that among these $N_{\text{test}}$ subjects, $N_{R}$ are found to be recent infections.

Note that there may be classification errors as the recency assay is unlikely to be perfect. To address this issue, Kassanjee et al. [3] proposed the following estimator for q=1:

$${\overset{ˆ}{\lambda }}_{0}=\frac{N_{R}-{\tilde{\beta }}_{T}N_{+}}{N_{-}\left({\tilde{\Omega }}_{T}-{\tilde{\beta }}_{T}T\right)}$$

Here $\Omega_{T}$ is the mean duration of recent infections (MDRI), and $\beta_{T}$ is the false recency rate (FRR), the probability of testing RITA-recent for a non-recently infected individual. ${\tilde{\Omega }}_{T}$ and ${\tilde{\beta }}_{T}$ are the estimates of these two parameters. MDRI and FRR are two important properties of a recency assay as they enable us to get an unbiased estimate the HIV incidence rate. Estimates of the assay parameters are commonly based on external studies of individuals that have known recent infections. For example, Kassanjee et al. [9] derived the estimates for a variety of recency assays based on the evaluation panel data of approximately 3,000 well-characterized subjects collected by the Consortium for the Evaluation and Performance of HIV Incidence Assays (CEPHIA).

### Standard error of Kassanjee’s estimator

In order to make inferences on the background HIV incidence, we also need to calculate the standard error of Kassanjee’s Estimator. This was developed in Gao et al. [4]. Here’s a brief re-capture of the model setup and results. Let p be the fraction of HIV-positive in the screening population. When q=1, i.e., $N_{\text{test}}=N_{+}$, Gao et al. [4] showed that under some equilibrium assumptions, we can model the data using the following multinomial model (Table 2):

Since we are assuming only a portion of the HIV-positive subjects will take the recency test, i.e., q may not be one, the above table will be slightly modified to the following.

The maximum likelihood estimator for $\lambda_{0}$ is given by

(1) $${\overset{ˆ}{\lambda }}_{0}=\frac{N_{R}N_{+}/N_{test}-{\tilde{\beta }}_{T}N_{+}}{N_{-}\left({\tilde{\Omega }}_{T}-{\tilde{\beta }}_{T}T\right)}$$

which reduces to the HIV incidence estimator proposed by Kassanjee et al. [3] when q=1. Gao et al. [4] gave the standard error formula of $log\;{\overset{ˆ}{\lambda }}_{0}$ when q=1, and in an earlier draft Gao et al. also gave the following standard error formula (squared) in general scenarios (q≤1):

$${\overset{ˆ}{V}}_{0}=\frac{N_{R}\left(N_{\text{test}}-N_{R}\right)}{N_{\text{test}}{\left(N_{R}-N_{\text{test}}{\tilde{\beta }}_{T}\right)}^{2}}+\frac{N}{N_{+}N_{-}}+{\tilde{\sigma }}_{\beta }^{2}\frac{N_{\text{test}}\left(N-N_{\text{test}}\right)}{N{\left(N_{R}-N_{\text{test}}{\tilde{\beta }}_{T}\right)}^{2}}+\frac{{\tilde{\sigma }}_{\Omega }^{2}}{{\left({\tilde{\Omega }}_{T}-{\tilde{\beta }}_{T}\right)}^{2}}\;\;+{\tilde{\sigma }}_{\beta }^{2}{\left\{\frac{N_{\text{test}}{\tilde{\Omega }}_{T}-N_{R}T}{\left(N_{R}-N_{\text{test}}{\tilde{\beta }}_{T}\right)\left({\tilde{\Omega }}_{T}-{\tilde{\beta }}_{T}\right)}\right\}}^{2}$$

where ${\tilde{\sigma }}_{\Omega }$ and ${\tilde{\sigma }}_{\beta }$ are the standard errors of ${\tilde{\Omega }}_{T}$ and ${\tilde{\beta }}_{T}$, respectively, obtained from evaluation panel data of external studies, for example in Kassanjee et al. [9]. The derivation of this formula is similar to the one in Gao et al. [4] and will not be repeated here.

### HIV PrEP trials based on recency assays and the Wald test

After we get the background HIV incidence estimates and the standard errors, we can use it as the counterfactual estimates to assess the efficacy of a PrEP agent in a HIV PrEP trial. The HIV Recency Assay Working Group proposed the following HIV PrEP trial design (see Figure 2 of [2]). In this design, during the screening process, subjects who are HIV-positive will take the recency test, and the background HIV incidence rate will be calculated using the Kassanjee’s Estimator. Subjects who are HIV-negative and meet other trial inclusion criteria will be enrolled in the active arm of the study and receive the intervention.

Among those HIV-negative subjects, suppose a random subset of $N_{\text{enroll}}$ subjects are recruited to the active arm of a clinical trial for the candidate PrEP agent and we observe $N_{\text{event}}$ incidence cases after an average of τ-years of follow-up. The efficacy of the candidate PrEP agent is determined by comparing the incidence of subjects receiving the candidate PrEP agent with the underlying incidence of HIV in enrolled participants in the absence of PrEP, i.e., the counterfactual placebo incidence of HIV that we denote as $\lambda_{0}$. For the active-arm trial, the number of events $N_{\text{event}}$ follows a Poisson distribution with mean $\lambda_{1}N_{\text{enroll}}\tau$, where $\lambda_{1}$ is the HIV incidence rate in the active arm. Write $R=\lambda_{1}/\lambda_{0}$ as the incidence ratio. The Wald test statistic is derived based on the standard errors of $log\overset{ˆ}{R}=log{\overset{ˆ}{\lambda }}_{1}-log{\overset{ˆ}{\lambda }}_{0}$ and is given by

$$Z=\frac{log\;{\overset{ˆ}{\lambda }}_{1}\;-\;log\;{\overset{ˆ}{\lambda }}_{0}\;-\;log\;R_{0}}{\sqrt{{\overset{ˆ}{V}}_{0}\;+\;{\overset{ˆ}{V}}_{1}}}$$

where ${\overset{ˆ}{V}}_{1}=1/N_{\text{event}}$. Under the null hypothesis, the Wald test statistic approximately follows a standard normal distribution.

## Likelihood ratio test and confidence intervals

The joint likelihood function for observed data related to the inference of candidate PrEP agent efficacy includes three parts: the probability for multinomial distribution in Table 3, the probability of the HIV infections in the active arm, and the probability of the observed recency assay parameters. For the first part, the log-likelihood can be written as

$$l_{1}\left(\lambda_{0},p,\Omega_{T},\beta_{T}\right)=\left(N_{\text{test}}-N_{R}\right)log\left[p\overset{ˆ}{q}-(1-p)\overset{ˆ}{q}\lambda_{0}\Omega_{T}-\left\{p-(1-p)\lambda_{0}T\right\}\overset{ˆ}{q}\beta_{T}\right]\;\;+N_{R}log\left[(1-p)\overset{ˆ}{q}\lambda_{0}\Omega_{T}+\left\{p-(1-p)\lambda_{0}T\right\}\overset{ˆ}{q}\beta_{T}\right]+\left(N_{+}-N_{\text{test}}\right)log\{p(1-\overset{ˆ}{q})\}\;\;+N_{-}log(1-p)$$

where $\overset{ˆ}{q}=N_{\text{test}}/N_{+}$.

For the second part, the log-likelihood can be written as

$$l_{2}\left(\lambda_{1}\right)=-\lambda_{1}N_{\text{enroll}}\tau +N_{\text{event}}log\left(\lambda_{1}N_{\text{enroll}}\tau \right)$$

For the third part, since the estimates and standard errors of MDRI and FRR are usually reported, and the MDRI and FRR are usually estimated independently, we assume the distribution of ${\tilde{\Omega }}_{T}$ and ${\tilde{\beta }}_{T}$ are independent, and approximate them by independent normal distributions, such that the log-likelihood is

$$l_{3}\left(\Omega_{T},\beta_{T}\right)=-\frac{{\left(\Omega_{T}\;-\;{\tilde{\Omega }}_{T}\right)}^{2}}{2{\tilde{\sigma }}_{\Omega }^{2}}-\frac{{\left(\beta_{T}\;-\;{\tilde{\beta }}_{T}\right)}^{2}}{2{\tilde{\sigma }}_{\beta }^{2}}+C$$

where C is a constant and can be ignored in the below calculations. The total log-likelihood is then

$$l\left(R,\lambda_{0},p,\Omega_{T},\beta_{T}\right)=l_{1}\left(\lambda_{0},p,\Omega_{T},\beta_{T}\right)+l_{2}\left(R\lambda_{0}\right)+l_{3}\left(\Omega_{T},\beta_{T}\right)$$

The maximum likelihood estimator (unconstrained) is given by

$$\left(\overset{ˆ}{R},{\overset{ˆ}{\lambda }}_{0},\overset{ˆ}{p},{\overset{ˆ}{\Omega }}_{T},{\overset{ˆ}{\beta }}_{T}\right)=\left(\frac{N_{event}}{{\overset{ˆ}{\lambda }}_{0}N_{enroll}\tau },{\overset{ˆ}{\lambda }}_{0},\frac{N_{+}}{N},{\tilde{\Omega }}_{T},{\tilde{\beta }}_{T}\right)$$

where ${\overset{ˆ}{\lambda }}_{0}$ is given in (1).

Under the null hypothesis $R=R_{0}$, the constraint maximum likelihood estimator will be obtained by maximizing $l\left(R_{0},\lambda_{0},p,\Omega_{T},\beta_{T}\right)$ over the four parameters ($\lambda_{0},p,\Omega_{T},\beta_{T}$), which can be numerically obtained by the R built-in function nlm(), or the nlpnms() subroutine in the IML procedure in SAS. For using the SAS procedure, a parameter transformation is needed for stable estimation such that we estimate $\left(\gamma_{1},\gamma_{2},\gamma_{3},\gamma_{4}\right)$ with $\gamma_{1}=log\left(\lambda_{0}\right),\;\gamma_{2}=log\{p/(1-p)\}$, $\gamma_{3}=log\left(\Omega_{T}-T\beta_{T}\right),\;\gamma_{4}=\beta_{T}$. For the initial values we used the unconstrained maximum likelihood estimates.

For two-sided p-values, we calculate the difference between the maximum log likelihood function and the maximum constrained log likelihood function given by

$$\Delta =\underset{R,\lambda_{0},p,\Omega_{T},\beta_{T}}{max}\;l\left(R,\lambda_{0},p,\Omega_{T},\beta_{T}\right)-\underset{\lambda_{0},p,\Omega_{T},\beta_{T}}{max}\;l\left(R_{0},\lambda_{0},p,\Omega_{T},\beta_{T}\right)$$

and the p-value will be calculated as $Pr\left(\chi_{1}^{2}\geq 2\Delta \right)$.

For one-sided p-values, we use the one-sided log-likelihood ratio test [11]. Specifically, we define

$$Z=sign(\overset{ˆ}{R}-R_{0})\sqrt{2\Delta }$$

which converges to a standard normal distribution. Here sign() is the usual sign function which takes value of 0, 1, −1, respectively, when the argument is 0, positive, negative. We then calculate the one-sided p-values based on Z.

Having developed the one-sided test, we can use the duality of hypothesis testing and interval estimation ([12], pp. 224–225) to derive the confidence interval. Specifically, the p-value obtained from the above procedure can be seen as a continuous decreasing function of $R_{0}$. And as $R_{0}$ varies from zero to infinity, the p-value will decrease from one to zero. To get the lower 1-α CI, we simply find an ${\overset{ˆ}{R}}_{L}$ such that the p-value is exactly α. In R this can be implemented using the built-in root-finding function uniroot(). The upper 1-α CI can be computed similarly.

## Simulations

In this section we evaluate the performance of the Wald test and the likelihood ratio test in realistic settings. Hypothetically we assume two trials will be conducted, one for men who have sex with men (MSM) or transgender women (TGW) in countries where the dominant HIV subtype is Subtype B, for example in Americas [13], and the other for cisgender women in Uganda, where the HIV subtypes are mixed and we assume the distribution is 45.5 % Subtype A, 2 % Subtype C, and 52.5 % Subtype D, based on Nazziwa et al. [14]. The simulations assumptions are listed in Table 4. Assume the Sedia LAg avidity assay will be used. The assay parameters and their relative standard errors (rSE) are from Kassanjee et al. [9] for the untreated people with an optical density normalized (ODn) cutoff of 1.5, and a viral load cutoff of 75 copies/mL, and infection cutoff time T of 2 years. The assumed HIV positivity rate of 5 % at screening is close to the 4 % positivity rate at screening for HPTN 083 [15] and HPTN 084 [8]. We consider the null hypothesis $H_{0}:R_{0}=0.5$, i.e., the active intervention is 50 % effective for preventing HIV infection for the cisgender women trial, and the null hypothesis $H_{0}:R_{0}=0.8$ for the MSM/TGW trial. We assume the target significance level is 0.025. For each simulation setting, we generated 10,000 independent samples, and the estimated true levels or power is calculated as the percentage of one-sided p-values that are <0.025 based on simulations generated from the null and alternative hypotheses, respectively.

One issue with the Wald test is on the handling the scenario of zero infections, as the Wald test would fail to give a p-value. We originally treated the Wald test p-value as non-significant (which we call the Wald test without correction) in the simulations for the following reasons:

1. The p-value can be seen as a continuous function of $N_{\text{event}}$. When $N_{\text{event}}$ approaches zero, the one-sided p-value for the Wald test converges to one, therefore it is more natural to treat it as non-significant.
2. Zero infections can be the result of small sample sizes, although it can also be an effect of a highly effective intervention. If we reject the null hypothesis whenever $N_{\text{event}}$ = 0, the power could increase as the sample size decreases, which does not make sense. Additionally the type I errors can be inflated for small sample sizes if we handle it in this way.

A referee pointed out that zero infections can be handled by the Wald test by adding a constant to the observed number of infections, usually 0.5. This technique has been employed by several authors albeit in slightly different contexts, e.g. in Price and Bonett [16] for ratio of two Poisson rates, and in Sweeting et al. [17] for odds ratios. We switched to this approach as the default in our simulations, and note it as the Wald test with corrections. We still presented some simulation results based on the Wald test without correction for comprehensiveness.

### Significance levels

**Figure 1:**
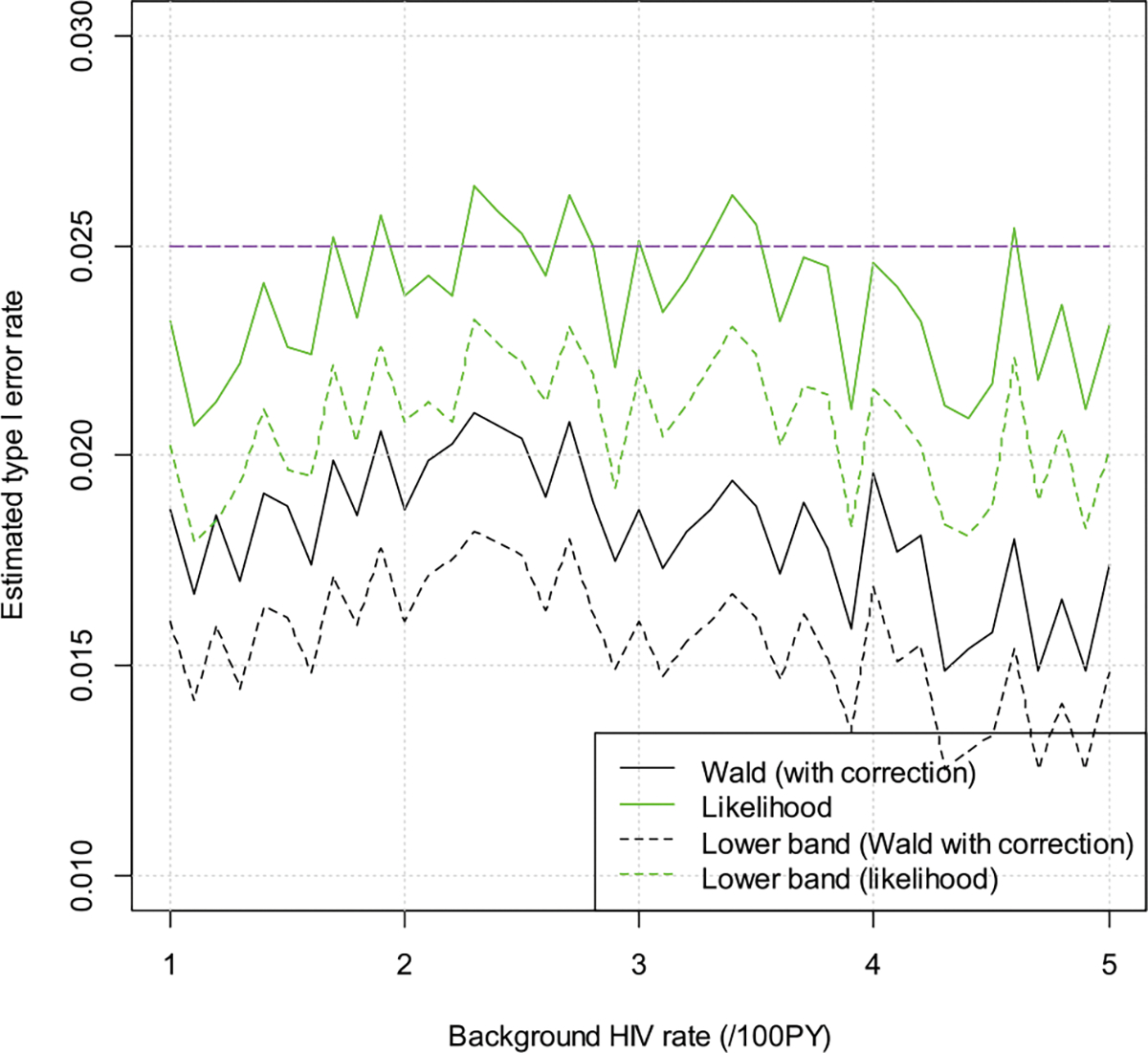
Estimated type I error rates for the cisgender women trial.

**Figure 2:**
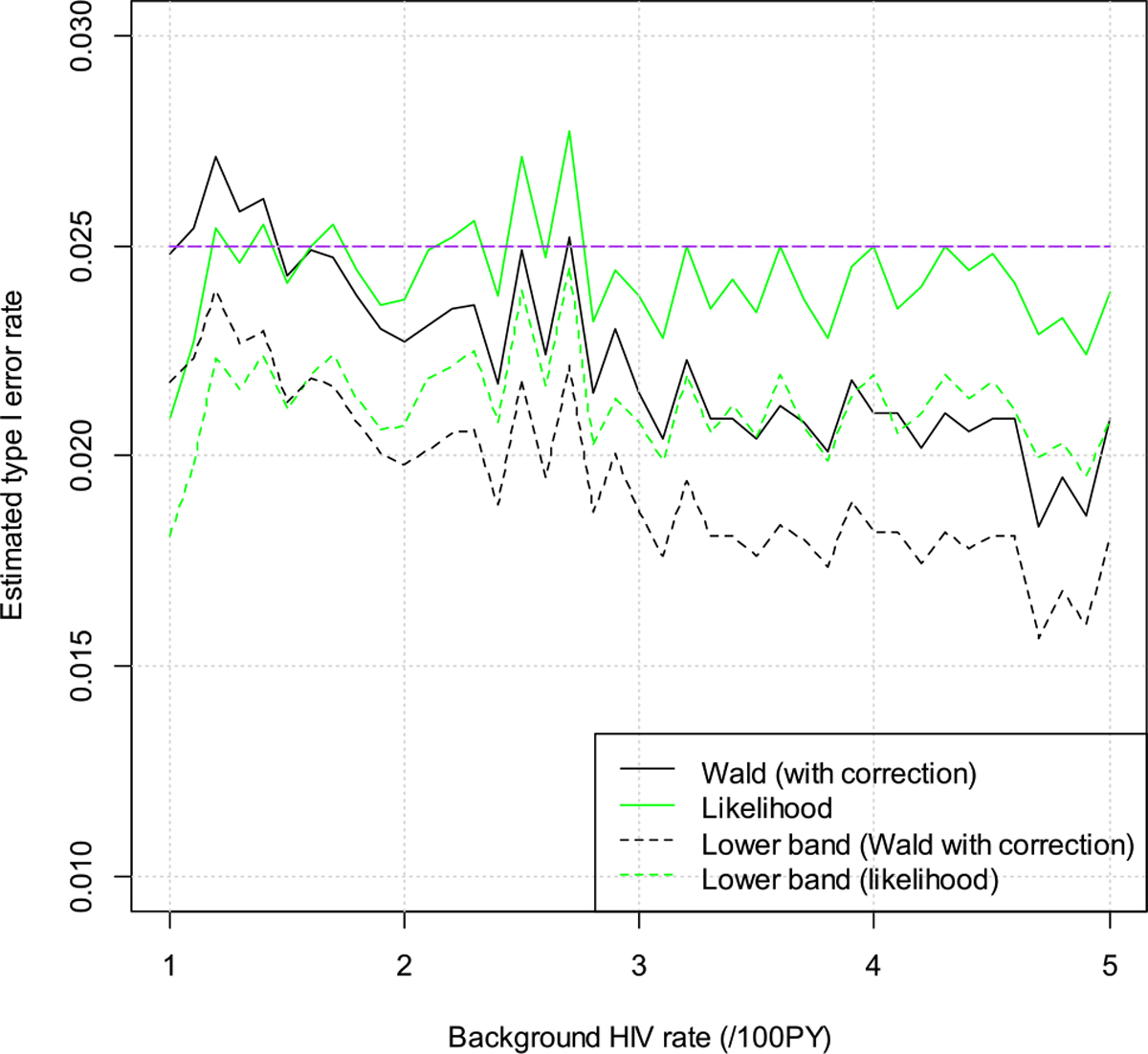
Estimated type I error rates for the men who have sex with men and transgender women (MSM/TGW) trial.

We assume null hypothesis is true, i.e., the risk ratios are 0.50 and 0.80, respectively, for the cisgender women and MSM/TGW trial. We vary the background HIV incidence rate from a low of 1 per 100 person years (/100PY) to a high of 5/100PY. The estimated significance levels are depicted in Figures 1 and 2. The purple line is for the target alpha of 0.025. The dashed lines are the lower confidence bands, i.e., the estimated significance levels minus 1.96 times the simulation standard of errors. From the plots we can see that both the Wald test and the likelihood ratio test control the type I error quite well. Occasionally the estimated type I error rate is above 0.025, but they are still within two times simulation standard error.

### Powers

**Figure 3:**
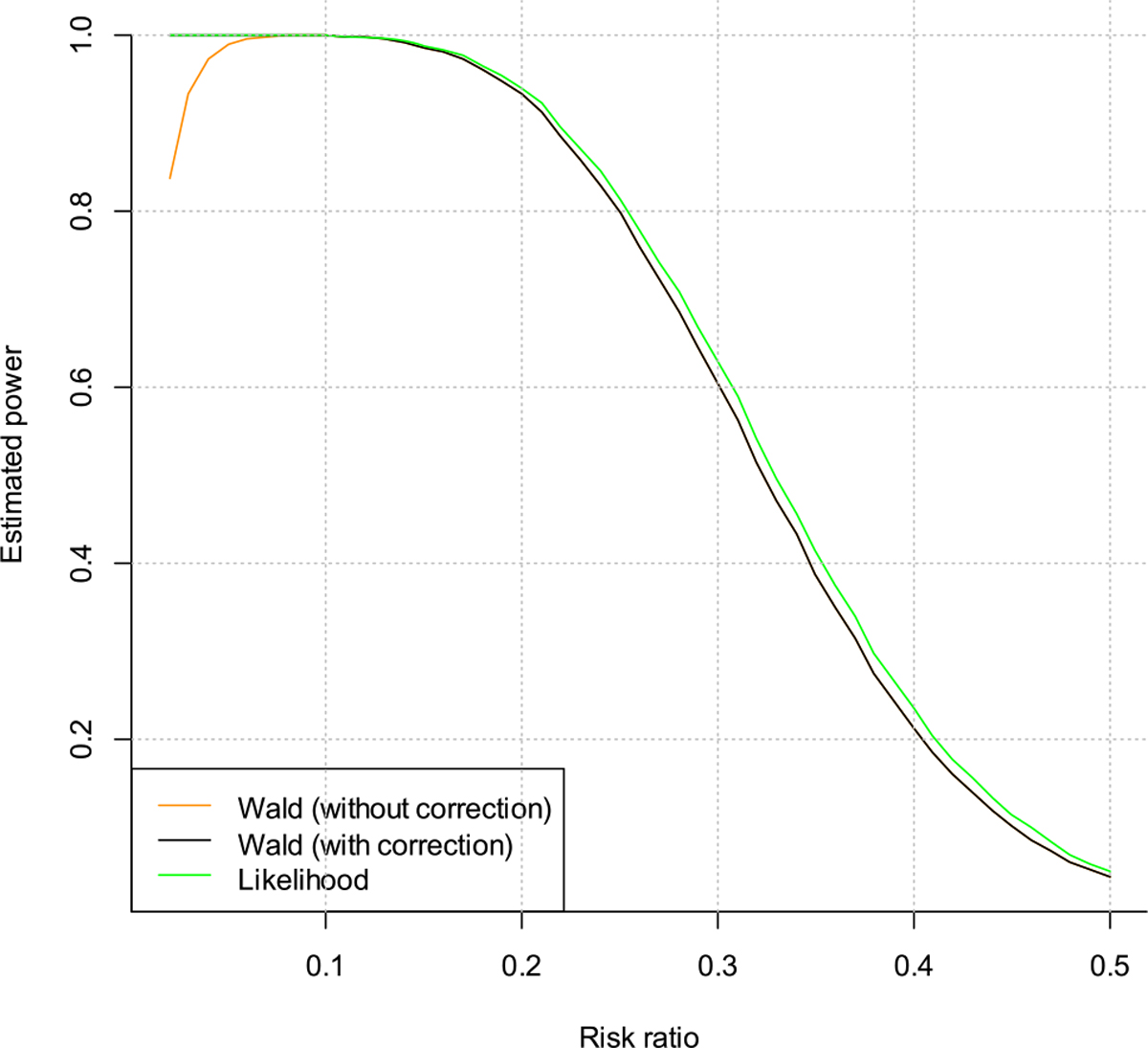
Estimated power vs. risk ratio for the cisgender women trial.

**Figure 4:**
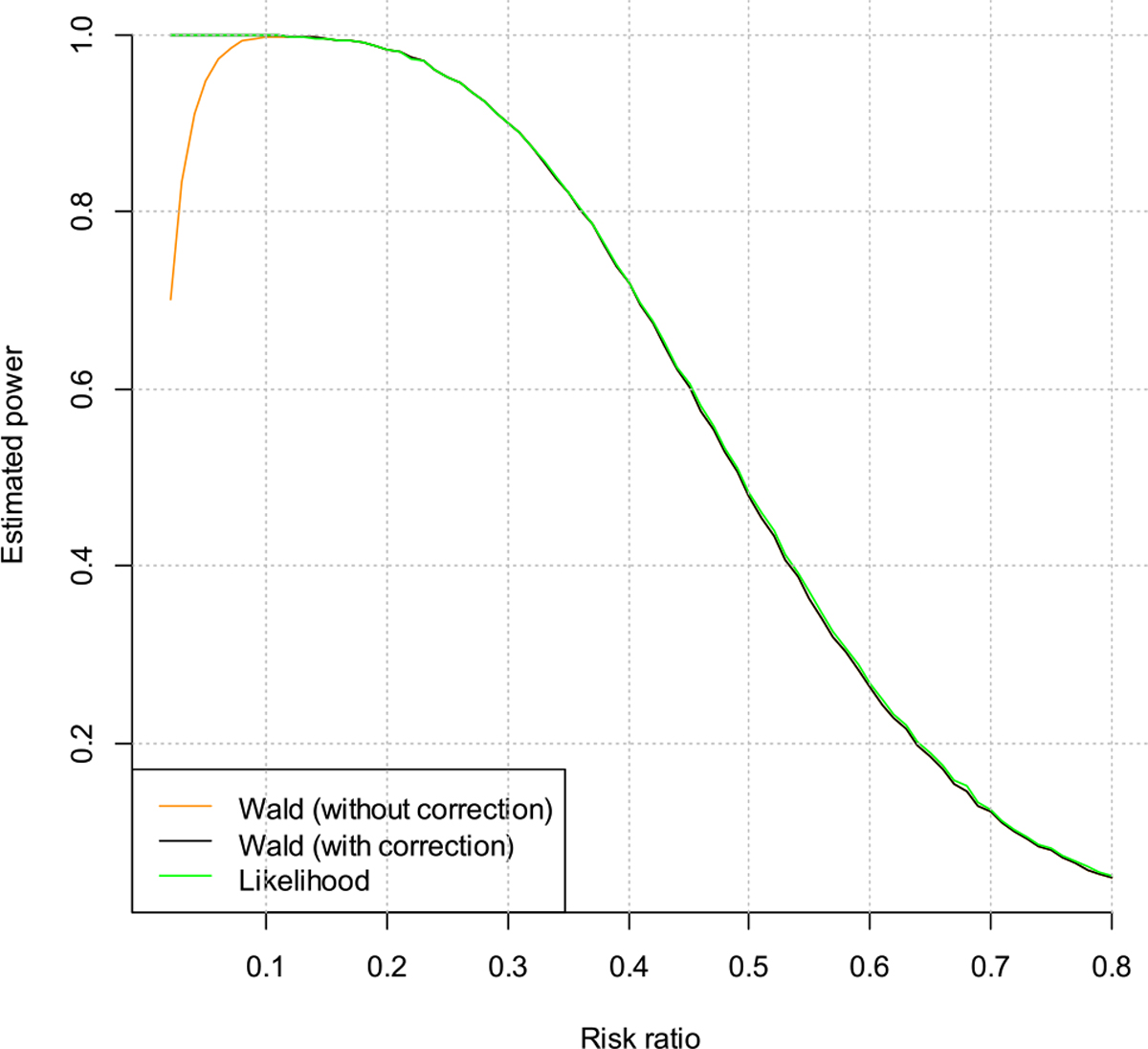
Estimated powers vs. risk ratio for the men who have sex with men and transgender women (MSM/TGW) trial.

For the power evaluations, we first fix the true background incidence rate at 3/100PY, and vary the risk ratio from 0.05 to 0.80. These values of risk ratio correspond to prevention efficacy of 95–20 % for the PrEP product evaluated in the trial. The estimated powers are plotted in Figures 3 and 4. Note that in Figure 4 the curve for the Wald test with correction is almost invisible because it almost perfectly coincides with the Wald test without correction when the risk ratio is >0.1. From the plots we can see that when the risk ratio is >0.10, both the Wald test and the likelihood ratio test have similar powers. But when the risk ratio is <0.10, the Wald test without correction has smaller power. Additionally, when the risk ratio decreases, i.e., the treatment effect becomes larger, the power for the Wald test without correction actually decreases. This is counter-intuitive.

We try to understand this phenomenon through taking a closer look to the Wald test statistic. When the risk ratio is very small, there is a higher chance that we will observe zero infections, in this case the Wald test would fail to give a p-value, and the Wald test without correction will treat it as non-significant, which decreases the power. This can be fixed by using the Wald test with correction, i.e., treating zero infections as 0.5 infection in the calculation. The likelihood ratio test does not have this issue and still performs properly in this case.

**Figure 5:**
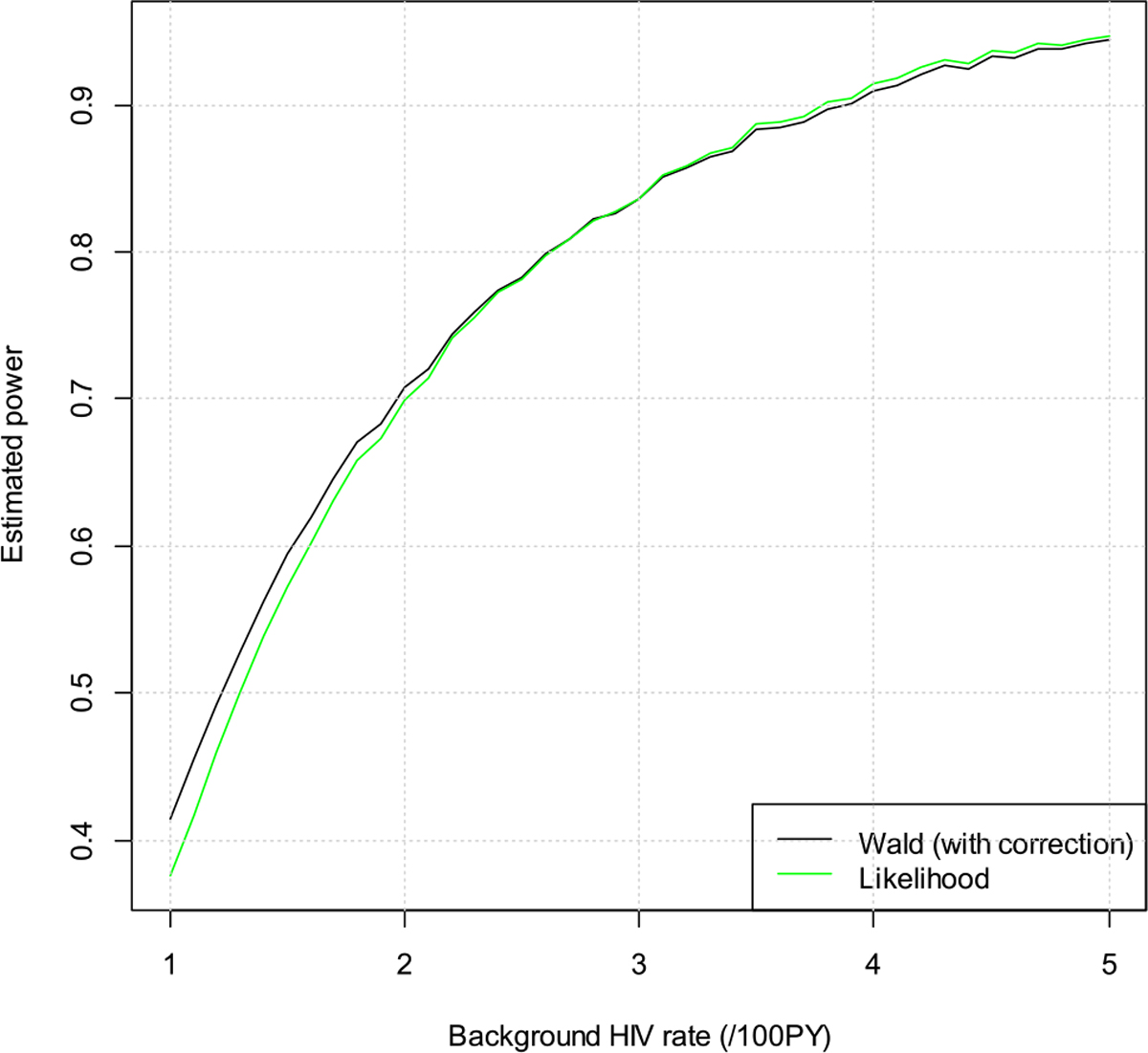
Estimated power vs. background HIV incidence rate for the men who have sex with men and transgender women (MSM/TGW) trial.

**Figure 6:**
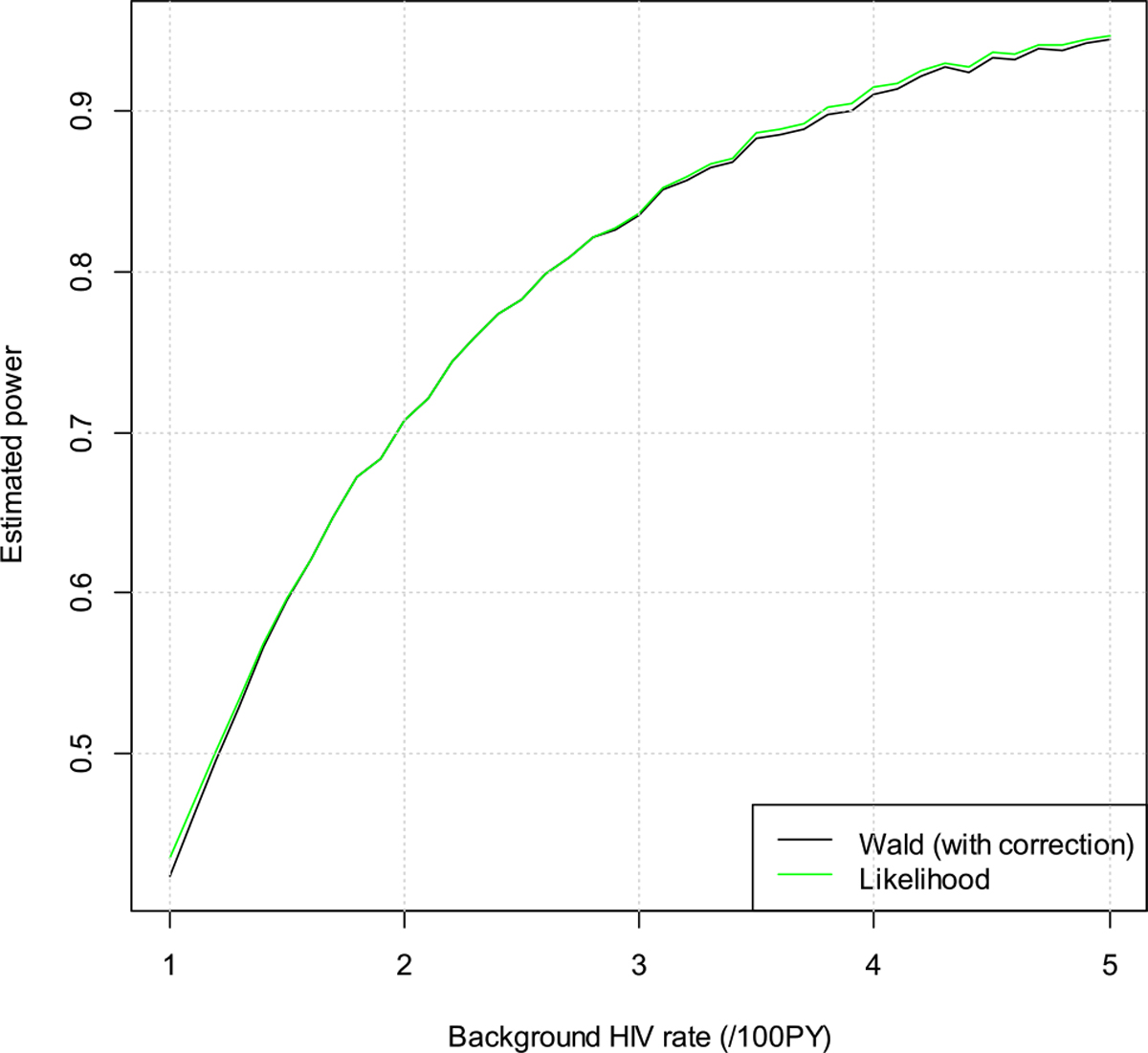
Estimated power vs. background HIV incidence rate for the men who have sex with men and transgender women (MSM/TGW) trial, assuming the relative standard error (rSE) of false recency rate (FRR) is 10 %.

We next fix the risk ratio at 0.3 and vary the background HIV incidence rate from 1/100PY to 5/100PY. The results for the MSM/TGW trial are in Figure 5. We can see that the Wald test has slightly higher power than the likelihood ratio test when the background HIV incidence rate is <2/100PY. The slight difference may be due to the slight deviations with the asymptotic results. In this example, the rSE of FRR is quite large at 98.7 %, and the deviations with the asymptotic results are therefore more noticeable. To verify this, we decrease the rSE of FRR to 10 %, and the results are in Figure 6. We can see that now the powers are similar for the two methods (differences are smaller than 0.02).

## Hypothetic example

Now let us consider a hypothetic example for the MSM/TGW trial where the dominant HIV subtype is Subtype B. Assume for the 3,000 screened participants, 5 % of them (150) tested positive for HIV, and 15 of these 150 were deemed recent infections by the RITA algorithm. The null hypothesis is *R*_0_ = 0.8. Suppose in the subgroup analysis or interim analysis we observe zero HIV infections in the active arm for a total duration of 3,000 person years, then we know that the Wald test would fail to work in this case, and we use 0.5 event in the calculation instead. The likelihood test still works in this case, and it gives a p-value (one-sided) of 0.00008, and a 95 % confidence interval for the risk ratio of (0, 0.058).

**Figure 7:**
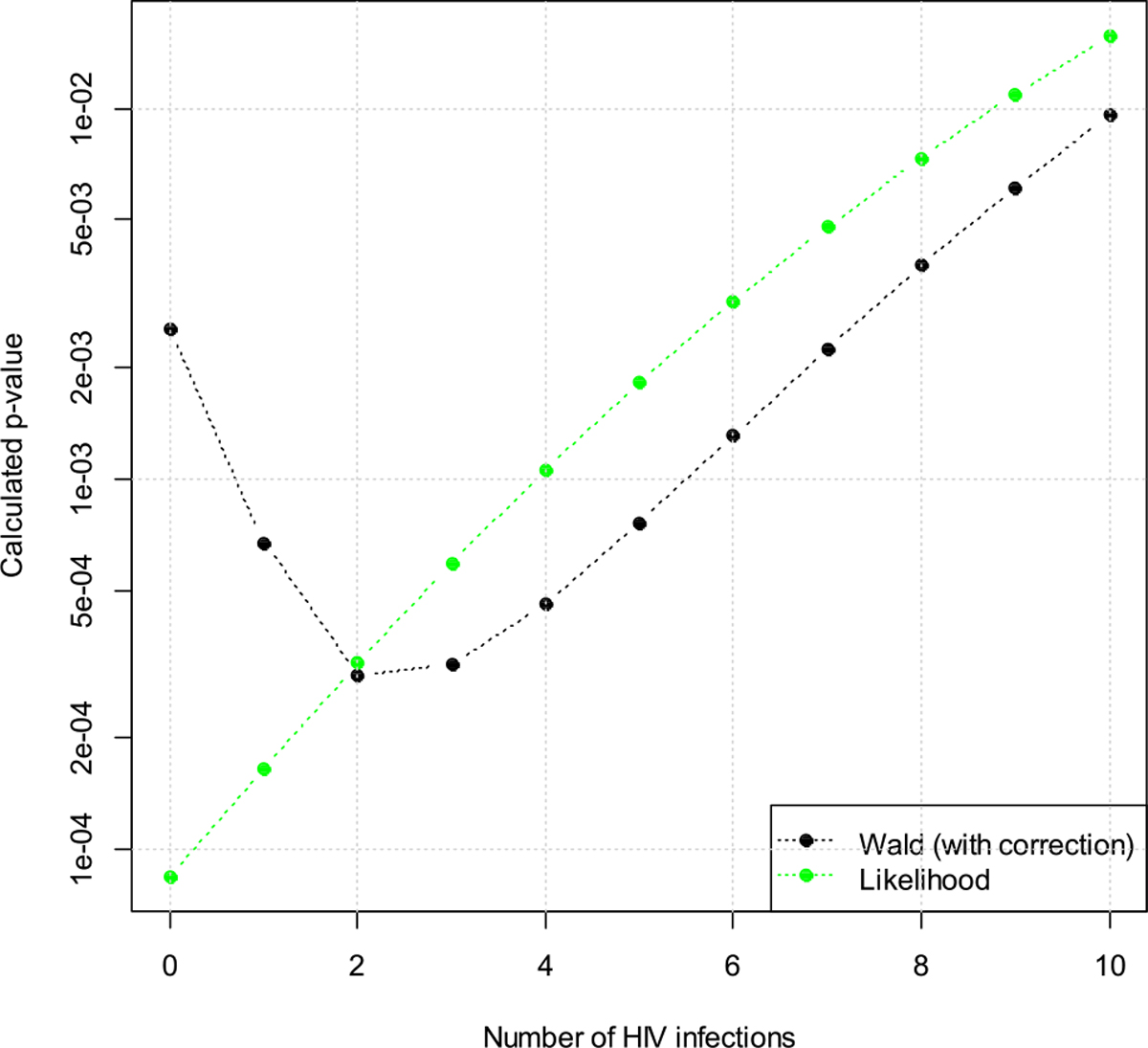
Hypothetic p-values (one-sided) from a trial for men who have sex with men and transgender women (MSM/TGW) when the number of HIV infections in the active arm is assumed to vary from zero to ten.

Next we vary the number of infections in the active arm from zero to ten. The resulting p-values (one-sided) are in Figure 7. We can see that the p-value is an increasing function with respect to the number of HIV infections, while the Wald test does not have this property.

We also considered a hypothetic example for the cisgender women trial in Uganda where the HIV subtypes are mixed. Assume for the 6,000 screened participants, 5 % of them (300) tested positive for HIV, and 15 of these 300 were deemed recent infections by the RITA algorithm. The null hypothesis is *R*_0_ = 0.8. Suppose we observe zero HIV infections in the active arm for a total duration of 3,000 person years. Again we know that the Wald test would fail to work in this case, and we use 0.5 event in the calculation instead. The likelihood test still works in this case and it gives a p-value (one-sided) of 0.014, and a 95 % confidence interval for the risk ratio of (0, 0.246).

**Figure 8:**
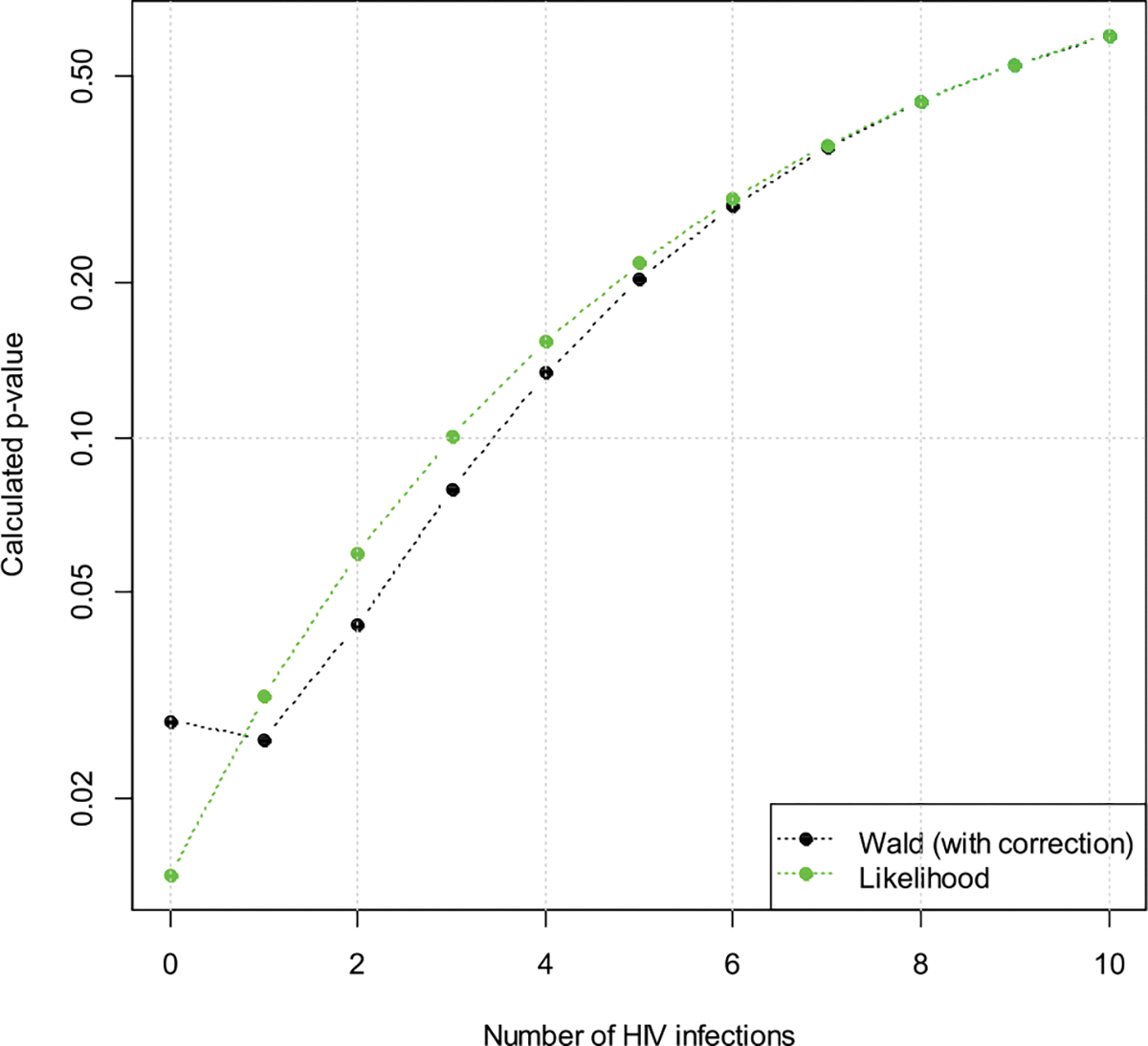
Hypothetical p-values (one-sided) for a trial in cisgender women when the number of infections in the active arm is assumed to vary from zero to ten.

Next we vary the number of infections in the active arm from zero to ten. The resulting p-values (one-sided) are in Figure 8. Again the p-value is an increasing function with respect to the number of HIV infections, while the Wald test does not have this property.

## Discussion

In this manuscript, we showed that the Wald test does not work when the number of infections is zero for HIV PrEP trials based on recency assay. The idea of adding 0.5 event when the number of infections is zero (or one) can solve this problem, but it still has the issue that more infections in the treatment phase could give smaller p-values. Additionally, adding 0.5 event could be seen as hard coding of trial data (which could artificially decrease the p-value). The very need of hard coding could invite scrutiny from regulatory agencies, as it could call into question the validity of the Wald test.

To solve this issue, we derived the likelihood ratio test and likelihood-based confidence interval for the HIV PrEP trials based on recency assay. Compared with the Wald test, it has the advantage that it works when there are zero HIV infections in the active arm, and the p-value is a monotone increasing function with respect to the number of infections in the active arm. Simulations were done to show that the likelihood ratio test controls the type I error well, and the adequate power can be achieved. We recommend using the likelihood-based tests and confidence intervals for HIV PrEP trials when the number of events in the active arm is small. For the currently ongoing HIV PrEP trials PURPOSE-1 and PURPOSE-2, the proposed method can be very useful especially in the subgroup analyses, as it can be expected that the number of HIV infections in some subgroups may be small and the Wald test may not perform well.

In the simulations, we evaluated the performance of the tests in the population with background incidence rate between 1/100PY and 5/100PY, as it is the population most likely to benefit from PrEP products. In many countries, background HIV incidence in the general population (i.e., not among members of key populations) is likely lower. However, conducting PrEP trials in such general population may not be feasible and thus is not considered in our simulation studies. For example, if we assume a 0.5/100PY background incidence rate, for the MSM/TGW trial, we would need 4,000 enrolled participants (and 6,000 enrolled participants if we also want to include an active control arm with 2:1 ratio) to get 80 % power for both the Wald test and the likelihood ratio test, assuming the true risk ratio is 0.2 and the null hypothesis is *R*_0_ = 0.8, and other settings being the same as Table 3. For the trial in cisgender women, if we use the setting in Table 3, we would need more than 20,000 enrolled participants for both the Wald test and the likelihood ratio test, assuming the true risk ratio is 0.2 and the null hypothesis is *R*_0_ = 0.5. Therefore, the sample size would be too large to be feasible with such a small background incidence rate.

## Figures and Tables

**Table 1:** Notations commonly used in active-arm trial with counterfactual incidence based on recency assay.

N	Number of volunteers screened for HIV
$N_{+}$	Number of HIV positive cases at screening
$N_{-}$	Number of HIV negative cases at screening. $N_{-}=N-N_{+}$
$N_{\text{test}}$	Number of HIV positive cases who take the recency test
$N_{R}$	Number of HIV positive cases identified as recent infections by the RITA algorithm
$N_{\text{enroll}}$	Number of HIV negative cases who are enrolled into the active arm
$N_{\text{event}}$	Number of HIV infections in the active arm
τ	Average follow-up time in the active arm
T	Infection cutoff time to define whether an infection is recent
$\Omega_{T}$	Mean duration of recent infection (MDRI) of the recency assay
$\beta_{T}$	False recency rate (FRR) of the recency assay

**Table 2:** Multinomial distribution of counts based on HIV and recency testing outcomes (*q* = 1).

Category	Count	Approximate probability

HIV+ (not recent)	$N_{+}-N_{R}$	$p-(1-p)\lambda_{0}\Omega_{T}-\left\{p-(1-p)\lambda_{0}T\right\}\beta_{T}$
HIV+ (recent)	$N_{R}$	$(1-p)\lambda_{0}\Omega_{T}+\left\{p-(1-p)\lambda_{0}T\right\}\beta_{T}$
HIV−	$N_{-}$	1-p

**Table 3:** Multinomial distribution of counts based on HIV and recency testing outcomes (*q* ≤ 1).

Category	Count	Approximate probability

HIV+ (not recent)	$N_{\text{test}}-N_{R}$	$pq-(1-p)q\lambda_{0}\Omega_{T}-\left\{p-(1-p)\lambda_{0}T\right\}q\beta_{T}$
HIV+ (recent)	$N_{R}$	$(1-p)q\lambda_{0}\Omega_{T}+\left\{p-(1-p)\lambda_{0}T\right\}q\beta_{T}$
HIV+ but not tested for recency	$N_{+}-N_{\text{test}}$	p(1-q)
HIV−	$N_{-}$	1-p

**Table 4:** Assumptions for the simulations.

Trial	Cisgender women	MSM/TGW

HIV subtype	Subtype A: 45.5 %	Subtype B
	Subtype C: 2 %	
	Subtype D: 52.5 %	
Infection cutoff time *T*, years	2	2
MDRI in days (rSE)	207(15.0 %)	146 (13.1 %)
FRR (rSE)	1.26 % (97.0 %)	1.3 % (98.7 %)
Number screened	6,000	3,000
Number enrolled	2,000	1,000
Average follow-up, years	1.5	2
Total follow-up (person years)	3,000	2,000
HIV positivity rate at screening	5%	5%
Null hypothesis	*R*_0_=0.5	*R*_0_=0.8

## Data Availability

Not applicable.
